# GATA3 inhibits GCM1 activity and trophoblast cell invasion

**DOI:** 10.1038/srep21630

**Published:** 2016-02-22

**Authors:** Yueh Ho Chiu, Hungwen Chen

**Affiliations:** 1 Graduate Institute of Biochemical Sciences, National Taiwan University, Taipei 106, Taiwan; 2 Institute of Biological Chemistry, Academia Sinica, Nankang, Taipei 115, Taiwan

## Abstract

Development of human placenta involves the invasion of trophoblast cells from anchoring villi into the maternal decidua. Placental transcription factor GCM1 regulates trophoblast cell invasion via transcriptional activation of HtrA4 gene, which encodes a serine protease enzyme. The GATA3 transcription factor regulates trophoblast cell differentiation and is highly expressed in invasive murine trophoblast giant cells. The regulation of trophoblastic invasion by GCM1 may involve novel cellular factors. Here we show that GATA3 interacts with GCM1 and inhibits its activity to suppress trophoblastic invasion. Immunohistochemistry demonstrates that GATA3 and GCM1 are coexpressed in villous cytotrophoblast cells, syncytiotrophoblast layer, and extravillous trophoblast cells of human placenta. Interestingly, GATA3 interacts with GCM1, but not the GCM2 homologue, through the DNA-binding domain and first transcriptional activation domain in GCM1 and the transcriptional activation domains and zinc finger 1 domain in GATA3. While GATA3 did not affect DNA-binding activity of GCM1, it suppressed transcriptional activity of GCM1 and therefore HtrA4 promoter activity. Correspondingly, GATA3 knockdown elevated HtrA4 expression in BeWo and JEG-3 trophoblast cell lines and enhanced the invasion activities of both lines. This study uncovered a new GATA3 function in placenta as a negative regulator of GCM1 activity and trophoblastic invasion.

The trophectoderm (TE) of peri-implantation embryo is the first differentiated linage for the development of trophoblast cell types in placenta. Human placenta is composed of villous tissues, which can be divided into floating villi immersed in maternal blood from uterine spiral arteries and anchoring villi attached to the maternal uterine decidua. The surface of placental villi contains a multinucleated syncytiotrophoblast (STB) layer, which is formed via cell-cell fusion of subjacent mononuclear cytotrophoblast (CTB) cells. CTB cells in the tip of anchoring villi proliferate and invade the decidua; they are called extravillous trophoblast (EVT) cells. These cells may replace the endothelial cells of spiral arteries enlarging their diameter for the promotion of maternal blood flow into the placenta. Murine trophoblast giant (TG) cells are equivalent to human EVTs^1^.

Human Glial Cells Missing-1 (GCM1) is a placenta-specific transcription factor expressed in villous CTB cells, the STB layer, and EVT cells^2^,^3^. At the molecular and cellular level, GCM1 upregulates the expression of syncytin-1 and syncytin-2 fusogenic proteins and HtrA4 serine protease to mediate trophoblast cell fusion and invasion, respectively^3^,^4^,^5^. Therefore, GCM1 is crucial for placental cell differentiation, which is corroborated by the fact that gene targeting of GCM1 results in embryonic death from defective placental development^6^,^7^. Intriguingly, GCM2, which is a GCM1 homologue, is essential for the development of the parathyroid gland^8^.

The GATA family of transcription factors is composed of six members, GATA1-6, with structural features of two N-terminal transactivation domains (TADs) and two carboxyl-terminal zinc fingers^9^. GATA3 is expressed in hematopoietic and nonhematopoietic tissues/cells such as mammary gland and T cells. In this scenario, GATA3 is crucial for T cell development, activation, proliferation, homeostasis, and effector functions^10^. Interestingly, GATA3 is abundantly expressed in the TG cells of mouse placenta. Along this line, GATA3 has been shown to regulate trophoblast stem cell differentiation and placental lactogen I and proliferin gene expression^11^.

As both GCM1 and GATA3 are key placental factors, we now investigate the possibility that both factors may interact with each other to regulate placental cell activity. Here we demonstrate that GATA3 physically interacts with GCM1, but not GCM2. Moreover, both factors are expressed in CTB cells, the STB layer, and EVT cells of first-trimester and term human placentas. By domain mapping analysis, we demonstrate that GCM1 and GATA3 interact with each other through the N-terminal DNA-binding domain (DBD) and the first TAD in GCM1 and the TADs and zinc finger 1 domain in GATA3. The interaction compromises the transcriptional activity of GCM1, but does not affect the DNA-binding activity of GCM1. As such the upregulation of HtrA4 promoter activity by GCM1 is impeded by GATA3, which further supports the observation that GATA3 knockdown enhances HtrA4 expression in BeWo and JEG-3 trophoblast cell lines and also the invasiveness of both lines. Our results reveal a novel function of GATA3 in control of trophoblast cell invasion through downregulation of GCM1 activity and HtrA4 expression.

## Results

### Expression of GATA3 and GCM1 in placenta

**Figure 1:**
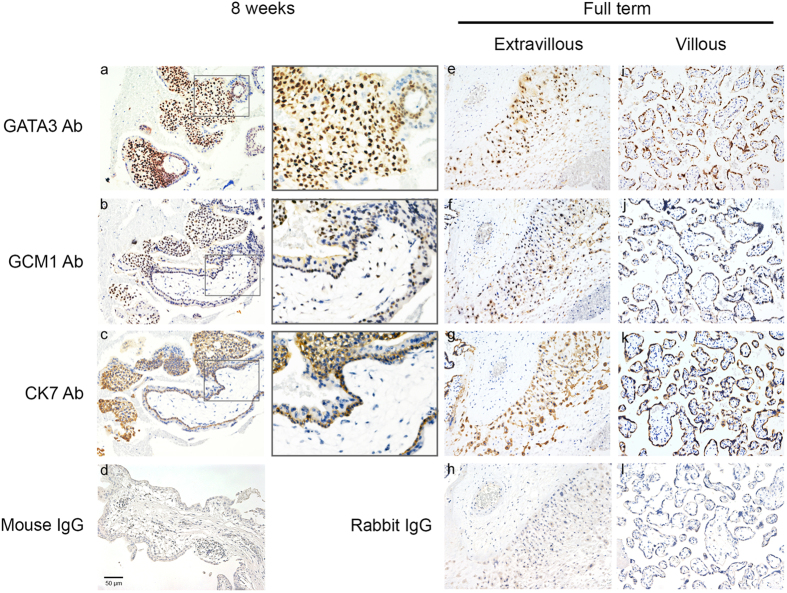
Expression of GATA3 and GCM1 in placenta. First-trimester (8 weeks, (**a**–**d**)) and full-term (**e**–**l**) placental sections were subjected to immunohistochemistry with mouse anti-GATA3 (**a**,**e**,**i**), rabbit anti-GCM1 (**b**,**f**,**j**), and mouse anti-cytokeratin 7 (CK7, (**c**,**g**,**k**)) Abs, respectively. In the first-trimester placenta, GATA3 and GCM1 are expressed in the CK7-positive trophoblast cells of cell column and villous cytotrophoblast cells. Magnified images of the boxed regions in first-trimester placental sections are shown on the right. Both GATA3 and GCM1 are highly expressed in the CK7-positive EVT cells and the CK7-positive syncytiotrophoblast layer of full-term placenta. As negative controls, first-trimester and full-term placental sections were incubated with normal mouse IgG (**d**) and normal rabbit IgG (**h**,**l**), respectively. Scale, 50 μm.

We investigated GATA3 and GCM1 expression in placenta by immunohistochemistry. The expression of the trophoblast marker, cytokeratin 7 (CK7), in placenta was also analyzed. In the first-trimester, both GCM1 and GATA3 were expressed in the nuclei of CK7-positive villous CTB cells and trophoblast cells of cell columns (Fig. 1, panels a–c). In the final stage of pregnancy, expression of both GCM1 and GATA3 was also detected in the nuclei of CK7-positive villous STB layer and EVT cells of full-term placentas (Fig. 1, panels e–g for EVT cells and i–k for STB layer). These observations suggested that GATA3 and GCM1 may be critical players in the control of trophoblast functions during pregnancy.

### GATA3 suppresses HtrA4 expression

**Figure 2:**
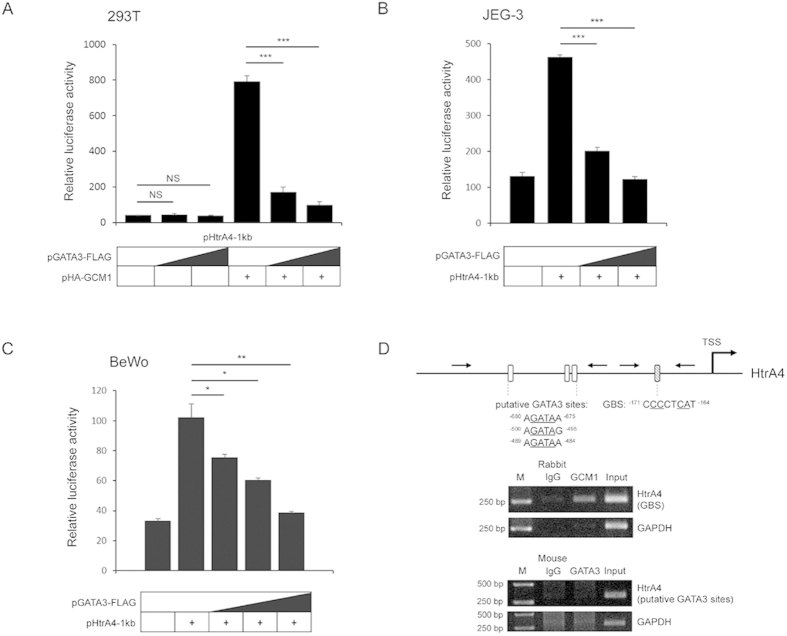
GATA3 downregulates HtrA4 promoter activity. 293 T (**A**) cells were transfected with 0.05 μg of pHtrA4-1 kb, 0.1 μg of pHA-GCM1, and increasing amounts of pGATA3-FLAG (0.1 and 0.3 μg), whereas JEG-3 (**B**) and BeWo (**C**) placental cells were transfected with pHtrA4-1 kb and increasing amounts of pGATA3-FLAG (0.1, 0.2, and 0.3 μg). At 48 h post-transfection, cells were harvested for luciferase reporter assays. (**D**) GCM1, but not GATA3, recognizes HtrA4 promoter. BeWo cells were subjected to ChIP analysis using GCM1 and GATA3 Abs, respectively. The immunoprecipitated complexes were analyzed by PCR with specific primer sets (arrows) flanking the GCM-binding site (GBS) or the putative GATA3 sites. Mean values and the S.D. obtained from three independent experiments are presented in (**A**–**C**).

Our recent study has indicated that the GCM1 target gene HtrA4 is expressed in EVT cells and may modulate EVT cell invasion^3^. Because both GATA3 and GCM1 are expressed in EVT cells, we were curious about whether GATA3 may regulate HtrA4 gene expression. We first examined the effects of GATA3 on GCM1-mediated transcriptional activation of HtrA4 promoter in transient expression experiments. As shown in Fig. 2A, GATA3-FLAG did not affect luciferase activity directed by HtrA4 promoter in the pHtrA4-1 kb reporter construct in 293 T cells, which lack endogenous GCM1 and GATA3 activity (Supplementary Figure 1). While HA-GCM1 alone stimulated the luciferase activity directed by pHtrA4-1 kb, coexpression of GATA3-FLAG counteracted the stimulatory effect of HA-GCM1 on pHtrA4-1 kb in a dose-dependent manner (Fig. 2A). We further performed similar experiments in JEG-3 and BeWo trophoblast cell lines, which exhibit endogenous GCM1 activity, and demonstrated that HtrA4 promoter activity is suppressed by increasing amounts of GATA3-FLAG (Fig. 2B,C). We also performed ChIP analyses in BeWo cells to test whether GATA3 recognizes potential GATA3 sites in the same HtrA4 promoter region assessed in pHtrA4-1 kb. Using a ChIP-grade GATA3 Ab, we failed to detect association of GATA3 with the HtrA4 promoter region harboring three putative GATA3 sites (Fig. 2D). As expected, GCM1 associated with the same assessed HtrA4 promoter region, which contains a previously-identified GCM1-binding site (GBS) (Fig. 2D). These results suggested that GATA3 may antagonize GCM1-mediated HtrA4 gene expression without binding to the promoter region of HtrA4 gene.

### GATA3 physically interacts with GCM1

**Figure 3:**
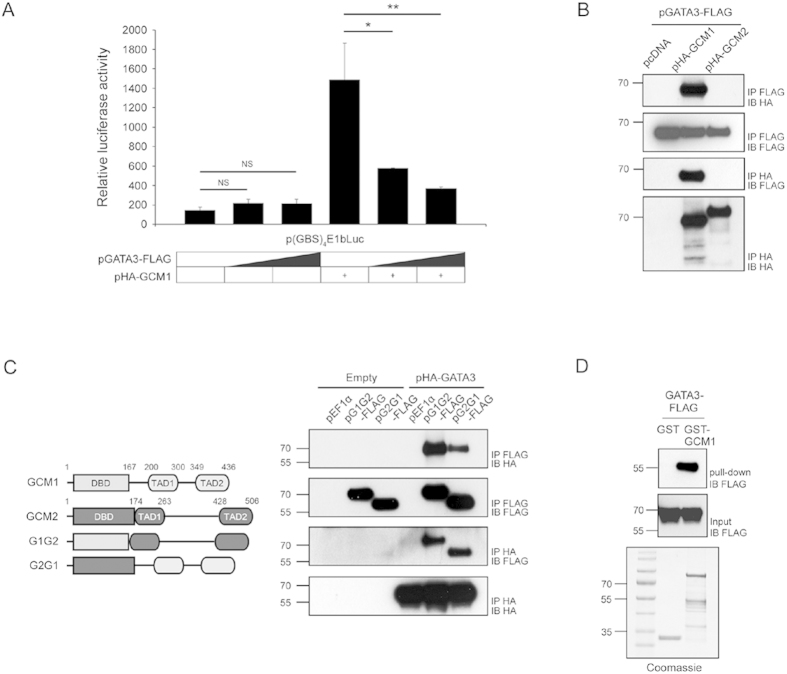
Physical interaction between GATA3 and GCM1. (**A**) GATA3 suppresses GCM1 transcriptional activity. 293 T cells were transfected with 0.05 μg of p(GBS)_4_E1bLuc, 0.1 μg of pHA-GCM1, and increasing amounts of pGATA3-FLAG (0.1 and 0.3 μg), followed by luciferase reporter assays. Mean values and the S.D. obtained from three independent experiments are presented. (**B**,**C**) GATA3 interacts with GCM1, but not GCM2. 293 T cells were transfected with 2.5 μg of pGATA3-FLAG and 2.5 μg of pHA-GCM1 or pHA-GCM2 (**B**). In a separate experiment, 293 T cells were transfected with 2.5 μg of pHA-GATA3 and 2.5 μg of pG1G2-FLAG or pG2G1-FLAG, which encodes a chimeric GCM1 and GCM2 protein (**C**). At 48 h post-transfection, cells were harvested for reciprocal coimmunoprecipitation assays with FLAG and HA Abs. (**D**) Direct interaction between GCM1 and GATA3. Approximately 45 ng of recombinant GATA3-FLAG protein was incubated with glutathione-conjugated agarose beads prebound with 0.5 μg of GST or 1.5 μg of GST-GCM1 in pull-down assays.

**Figure 4:**
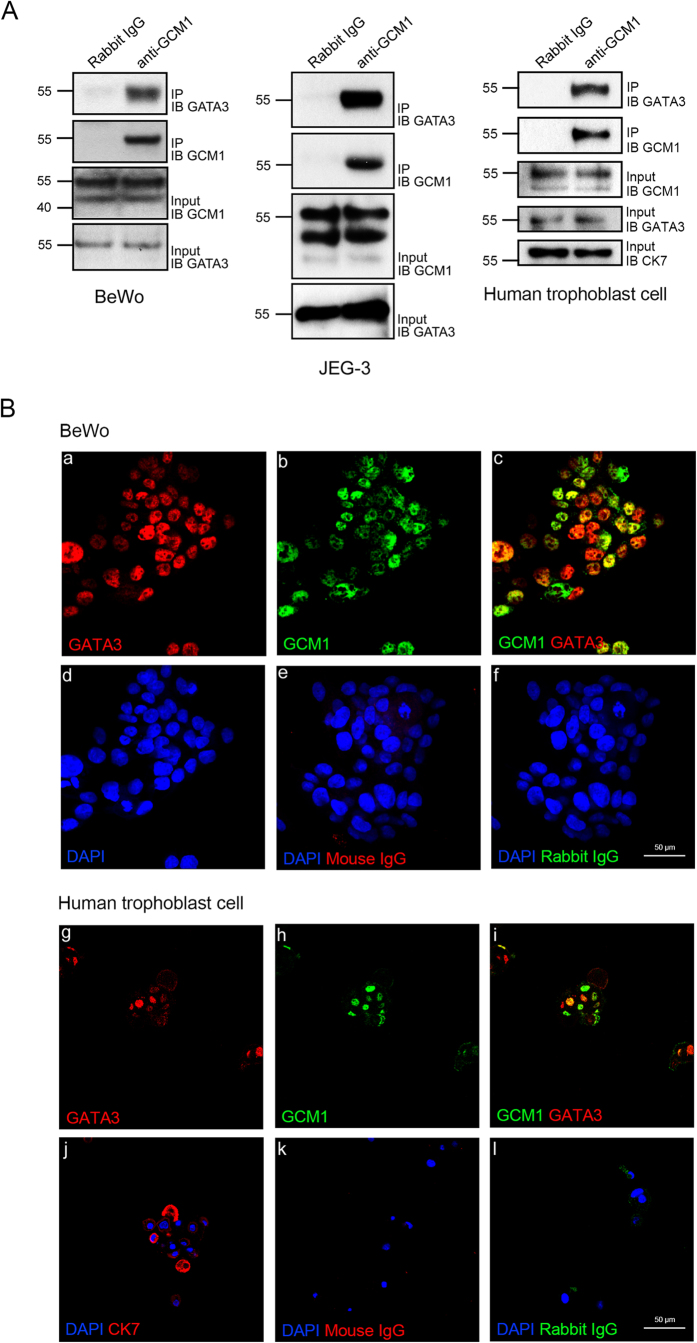
Interaction and co-localization of GATA3 and GCM1 in placenta. (**A**) Interaction of endogenous GATA3 and GCM1 in placental cells. BeWo, JEG3, and primary human trophoblast cells were subjected to coimmunoprecipitation assays with rabbit normal IgG or rabbit anti-GCM1 Ab for immunoprecipitation and mouse anti-GATA3 Ab for immunoblotting. Input controls are provided for GCM1, GATA3, and CK7 (human trophoblast cells) in the lower panels. (**B**) Immunofluorescence microscopy of GATA3 and GCM1 in placental cells. BeWo (a–f) and primary human trophoblast (g–l) cells were fixed, permeabilized, and stained with mouse anti-GATA3 (a,g), rabbit anti-GCM1 (b,h), and mouse anti-CK7 (j) Abs. Cells were then incubated with secondary Abs and examined under a confocal microscope. Panels c and i are the merge images of GATA3 and GCM1 staining in BeWo (a,b) and human trophoblast (g,h) cells, respectively. Nuclei were stained with DAPI (blue, d–f,j–l). As negative controls, cells were stained with normal mouse IgG (e,k) and normal rabbit IgG (f,l). Scale, 50 μm.

We speculated that GATA3 may physically interact with GCM1 to antagonize the transcriptional activity of GCM1. To test this hypothesis, we first examined the effect of GATA3 on GCM1 transcriptional activity using a GCM1-specific reporter construct, p(GBS)_4_E1bLuc. As shown in Fig. 3A, GATA3-FLAG significantly suppressed the transcriptional activity of HA-GCM1 in 293 T cells cotransfected with pHA-GCM1 and increasing amounts of pGATA3-FLAG. Subsequently, we preformed reciprocal coimmunoprecipitation assays in 293 T cells cotransfected with pHA-GCM1 and pGATA3-FLAG and detected specific interaction between HA-GCM1 and GATA3-FLAG (Fig. 3B). As a control, coexpression of GATA3-FLAG with the HA-tagged GCM2 homologue revealed no interaction between GATA3 and GCM2 (Fig. 3B). By domain swapping analysis, we further demonstrated that the DBD or TAD of GCM1 confers GATA3-binding activity to DBD or TAD of GCM2 (Fig. 3C). Direct physical interaction between GCM1 and GATA3 was supported by pull-down assays in which recombinant GATA3-FLAG bound to GST-GCM1, but not GST (Fig. 3D). Specific interaction between endogenous GCM1 and GATA3 in BeWo, JEG-3, and primary human trophoblast cells was confirmed by coimmunoprecipitation assays with rabbit anti-GCM1 Ab for immunoprecipitation and mouse anti-GATA3 Ab for immunoblotting (Fig. 4A). Furthermore, immunofluorescence microscopy revealed co-localization of endogenous GCM1 and GATA3 in BeWo and primary human trophoblast cells (Fig. 4B, panels c,i). Collectively, these results suggested that GATA3 directly interacts with GCM1 and inhibits the transcriptional activity of GCM1 in placenta.

### Characterization of Interaction Domains of GCM1 and GATA3

**Figure 5:**
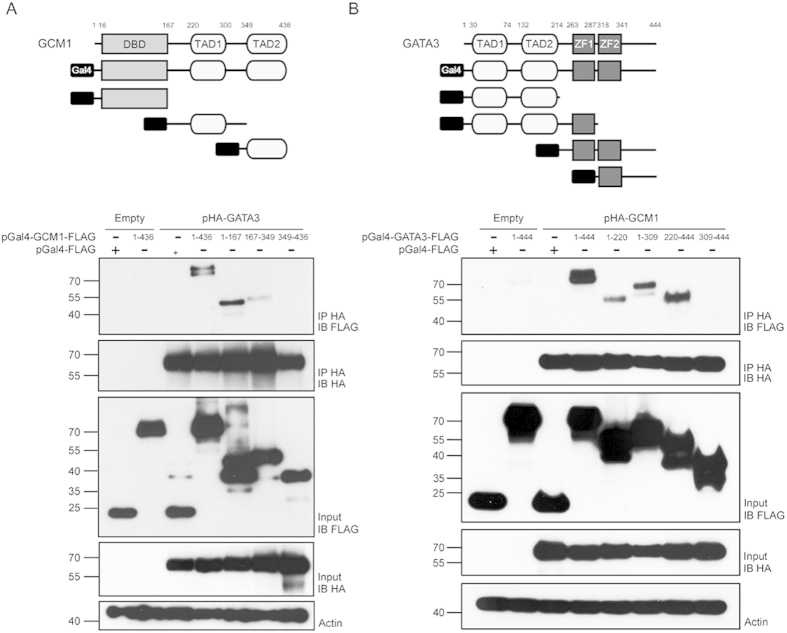
Analysis of interaction domains in GCM1 and GATA3. (**A**) Mapping of GATA3-interacting domains in GCM1. 293 T cells were transfected with 2.5 μg of pHA-GATA3 and a serial of pGal4-GCM1-FLAG constructs (2.5 μg) encoding Gal4 fusion proteins with full-length GCM1 or different GCM1 domains. (**B**) Mapping of GCM1-interacting domains in GATA3. 293 T cells were transfected with 2.5 μg of pHA-GCM1 and a series of pGal4-GATA3-FLAG constructs (2.5 μg) encoding Gal4 fusion proteins with full-length GATA3 or different GATA3 domains. At 48 h post-transfection, cells were harvested for coimmunoprecipitation assays with HA and FLAG Abs.

To further characterize the interaction between GCM1 and GATA3, we mapped the interaction domains of GCM1 for GATA3, and vice versa. Coimmunoprecipitation assays were conducted in 293 T cells cotransfected with pHA-GATA3 and pGal4-FLAG or pGal4-GCM1-FLAG harboring the full-length GCM1 or truncated GCM1 domains. As shown in Fig. 5A, strong interaction was detected between HA-GATA3 and full-length GCM1 and the N-terminal domain (amino acids 1–167) of GCM1. In addition, modest interaction was detected between HA-GATA3 and the region of amino acids 167–349 in GCM1, which constitutes the first TAD (TAD1) of GCM1 (Fig. 5A). To map the interaction domain of GATA3 for GCM1, 293 T cells were cotransfected with pHA-GCM1 and pGal4-FLAG or pGal4-GATA3-FLAG harboring the full-length GATA3 or truncated GATA3 domains for coimmunoprecipitation assays. Interaction was detected between HA-GCM1 and full-length GATA3 and GATA3 truncated mutants harboring the N-terminal TADs or the first zinc finger 1 (ZF1) domain (Fig. 5B). We also generated ZF1-deletion mutant GATA3 constructs, pGal4-GATA3ΔZF1-FLAG and pGATA3ΔZF1-FLAG, and demonstrated that Gal4-GATA3ΔZF1-FLAG is able to interact with HA-GCM1 and that GATA3ΔZF1-FLAG suppresses the transcriptional activity of HA-GCM1 in transient expression experiments (Supplementary Figure 2). Therefore, the N-terminal TADs in ZF1-deletion mutant GATA3 are very likely responsible for interaction with HA-GCM1.

### GATA3 inhibits the activity of GCM1 TAD

**Figure 6:**
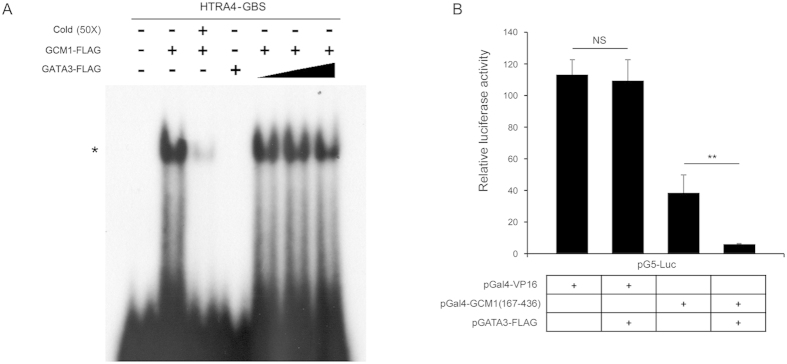
GATA3 blocks GCM1 transcriptional activity. (**A**) GATA3 does not affect the DNA-binding activity of GCM1. Approximately 20 ng of recombinant GCM1-FLAG alone or plus increasing amounts of GATA3-FLAG (20, 40, and 60 ng) was incubated with 2 ng of radiolabeled HtrA4-GBS probe in the EMSA analysis. The asterisk indicates the GCM1-FLAG-DNA complex. Of note, 20 ng of recombinant GATA3-FLAG alone did not recognize the probe. (**B**) Suppression of the activity of GCM1 TAD by GATA3. 293 T cells were transfected with different combinations of 0.05 μg of pG5-Luc, 0.1 μg of pGal4-VP16, 0.1 μg of pGal4-GCM1(167–436), and 0.3 μg of pGATA3-FLAG. At 48 h post-transfection, cells were harvested for luciferase reporter assay. Mean values and the S.D. obtained from three independent experiments.

The suppressive effect of GATA3 on GCM1 activity can be attributed to inhibition of the activity of GCM1 DBD and/or TAD by GATA3. To test whether GATA3 interferes with the DNA-binding activity of GCM1, EMSA analysis was performed using recombinant GCM1-FLAG and GATA3-FLAG proteins and a radiolabeled HtrA4 GBS (HtrA4-GBS) probe. As shown in Fig. 6A, the HtrA4-GBS probe was specifically recognized by GCM1-FLAG, but not GATA3-FLAG. Importantly, the binding of GCM1-FLAG to HtrA4-GBS was not affected by increasing amounts of GATA3-FLAG (Fig. 6A). To test the effect of GATA3 on the transcriptional activity of GCM1, the GCM1 TAD (amino acids 167–436) was fused with the Gal4 DBD [Gal4-GCM1(167–436)]. We cotransfected 293T cells with pG5-Luc reporter construct, which harbors five tandem copies of Gal4-binding site, and different combinations of pGATA3-FLAG, pGal4-VP16, and pGal4-GCM1(167–436). As shown in Fig. 6B, the stimulation of luciferase reporter gene expression by Gal4-GCM1(167–436) was significantly suppressed by GATA3-FLAG. Taken together, these results suggested that GATA3 suppresses GCM1 activity through inhibition of its TAD activity.

### GATA3 negatively regulates HtrA4 expression and placental cell invasion

**Figure 7:**
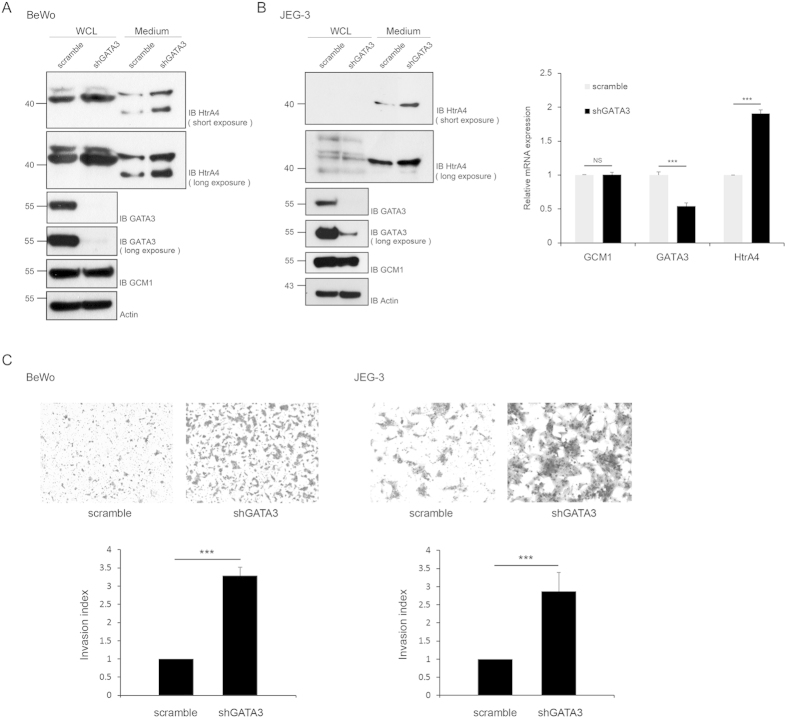
Regulation of placental cell invasion by GATA3. (**A**,**B**) GATA3 knockdown elevates HtrA4 expression in BeWo and JEG-3 cells. Culture media and whole cell lysates (WCLs) were harvested from BeWo and JEG-3 cells stably expressing scramble or GATA3 shRNA (shGATA3) for immunoblotting analysis with the indicated Abs. In a separate experiment, the scramble and GATA3 shRNA-expressing JEG-3 cells were harvested for quantitative real-time PCR analysis for GCM1, GATA3, and HtrA4 transcripts, respectively. (**C**) GATA3 knockdown enhances placental cell invasion. BeWo and JEG-3 cells stably expressing scramble or GATA3 shRNA were plated in Matrigel-coated chambers for 24 h. The invasive BeWo and JEG-3 cells in the lower surface of the filters were fixed, stained, and counted. Mean values and the S.D. obtained from three independent experiments are presented in (**B**,**C**).

We now investigated the effects of GATA3 on GCM1-regulated cellular activity in terms of cell invasion. As HtrA4 is a GCM1 target gene modulating placental cell invasion, HtrA4 expression was analyzed in BeWo and JEG-3 cells expressing scramble or GATA3 shRNA. Knocking down GATA3 in both BeWo and JEG-3 cells increased the HtrA4 protein level in culture media and whole cell lysates compared with that of control cells expressing scramble shRNA (Fig. 7A,B). Correspondingly, the HtrA4 transcript level was upregulated by knocking down GATA3 as measured in the GATA3-knockdown JEG-3 cells (Fig. 7B). The invasion activity of GATA3-knockdown BeWo and JEG-3 cells was measured by Matrigel-coated transwell assay. Compared with the control cells expressing scramble shRNA, the invasion activities of GATA3-knockdown BeWo and JEG-3 cells were significantly enhanced (Fig. 7C).

## Discussion

GATA switch between GATA1 and GATA2 has been reported to drive differentiation of blood cell lineages from hematopoietic progenitors^12^. By analogy, the replacement of Gata3 with Gata2 in the GATA sites of Gata2 promoter has been reported to stimulate the differentiation of rodent Rcho-1 trophoblast cells into TG cells^13^. Here we provided evidence to support an additional function of GATA3 in regulation of trophoblast cell invasion via protein-protein interaction with the placenta-specific GCM1 transcription factor. Specifically, GATA3 interacts with GCM1 and inhibits GCM1-mediated HtrA4 gene activation to downregulate the invasion activity of trophoblast cells.

Although several potential GATA3 sites were found in the first 1 kb region of HtrA4 promoter, their involvement in the suppression of HtrA4 gene expression by GATA3 was not detected in the present study. This notion was also substantiated by the facts that HtrA4 promoter activity is not affected by increasing amounts of GATA3 in transient expression experiments (Fig. 2A) and that ChIP analysis fails to detect the association between GATA3 and HtrA4 promoter (Fig. 2D). Instead, we favored the model that GATA3 suppresses HtrA4 expression by impairing the transcriptional activity of GCM1 based on the following observations. First, GATA3 suppresses the transcriptional activation of the pG5-Luc reporter construct by Gal4-GCM1(167–436), which is the fusion protein of Gal4 BDB and GCM1 TAD. Second, GATA3 directly interacts with GCM1 (by pull-down assay). Third, GATA3 does not affect the binding of GCM1 to the GBS of HtrA4 gene (by EMSA analysis).

The GCM2 transcription factor, a GCM1 homologue, is essential for the development of parathyroid gland^8^. Recently, Han *et al.*^14^ have reported that mouse Gata3 may collaborate with SP1, Gcm2, and MafB to upregulate the promoter activity of human parathyroid hormone (PTH) gene. A GC-box, instead of a putative GATA3 site in the proximal PHT promoter region was required for Gata3, Gcm2, and MafB to activate PTH promoter. Potential interaction between overexpressed Gata3 and Gcm2 was implicated by coimmunoprecipitation analysis, yet we also noticed that the PTH promoter activity stimulated by Gata3 was significantly compromised when Gcm2 was coexpressed. This functional antagonism between Gata3 and Gcm2 is similar to that between GATA3 and GCM1 in the present study. Moreover, we clearly demonstrated that the functional antagonism between GATA3 and GCM1 in regulation of HtrA4 gene expression is most likely due to direct interaction between the two factors in placenta. It remains unclear whether Gata3 directly interacts with Gcm2 and whether endogenous Gata3 and Gcm2 interact with each other in parathyroid precursor cells. In contrast to the aforementioned study of mouse Gata3 and Gcm2, we found that human GATA3 does not interact with human GCM2 when coexpressed in 293 T cells. Interestingly, domain swapping experiments with chimeric GCM1 and GCM2 constructs showed that GCM1 DBD and TAD domains confer the GATA3-binding activity to GCM2 DBD and TAD domains. Given that both GCM1 and GCM2 share 66.9% polypeptide sequence identity in their DBDs (also named as the GCM motif), no sequence homology can be detected in their TADs. Nevertheless, the configuration of TADs is different between GCM1 and GCM2 such that the TAD1 is close to the DBD and away from the TAD2 in GCM2 (Fig. 3C). An inhibitory domain, which affects protein stability and transcriptional activity, has been identified in the region flanked by the two TADs of GCM2^15^. The possibility that intramolecular interaction between DBD and TADs in GCM2 blocks its interaction with GATA3 cannot be ruled out. Further structural investigation is needed to elucidate differential GATA3-binding activity between GCM1 and GCM2.

GATA3 regulates the differentiation of CD4^+^ T cells into T helper 2 (Th2) by suppression of STAT4, IL-18Rα, and IL-12Rβ2 gene expression, which is crucial for INF-γ production^10^. Besides, GATA3 may interact with RUNX3 and blocks RUNX3-mediated INF-γ gene expression, which occurs during the differentiation of Th1 cells^16^. On the other hand, the ASH2L component of the MLL histone methyltransferase complex for H3K4 methylation has recently been shown to interact with GATA3 and enhance GATA3-mediated ERα gene expression in breast cancers^17^. Therefore, GATA3 may positively or negatively regulate a variety of cellular activities depending on its interacting proteins. While Gata3 activates Cdx2 and placental lactogen I expression in trophoblast stem cells and Rcho-1 cells, respectively^13^,^18^, the physical and functional interaction between GATA3 and GCM1 in the present study has further revealed a new function of GATA3 in the regulation of human placental cell invasion.

## Methods

### Plasmid constructs

The HA-tagged GCM1 expression plasmid, pHA-GCM1, has been described previously^19^. A DNA fragment encoding human GATA3 with an N-terminal triple HA tag or a C-terminal triple FLAG tag was subcloned into a CMV or EF1 promoter-driven expression vector to generate pHA-GATA3 or pGATA3-FLAG. The HtrA4 promoter region from nt −971 to 29 (relative to the translational initiation site) was subcloned into pGL3E1B to generate the pHtrA4-1 kb reporter plasmid. The p(GBS)_4_E1bLuc reporter plasmid was generated by subcloning four copies of the proximal GCM1-binding site derived from the syncytin-1 gene in the pGL3E1B luciferase reporter vector. The pG5-Luc reporter plasmid which harbors five copies of the Gal4-binding site was obtained from Promega (Madison, WI).

### Cell culture, transfection, and lentivirus transduction

293 T, BeWo, and JEG-3 cells were obtained from the American Type Culture Collection (Manassas, VA) and maintained at 37 °C in minimal essential medium alpha medium (for 293 T and JEG-3) or F-12 K medium (for BeWo) supplemented with fetal bovine serum, streptomycin, and penicillin. Placental villous tissues were obtained from term placentas of healthy women undergoing elective cesarean section with informed consent according to the study protocol (CT-100058) approved by the Institutional Review Board of Cathay General Hospital of Taiwan. Primary human trophoblast cells were prepared from normal term placentas according to Kliman *et al.*^20^ and cultured at 37 °C in Iscove’s modified Dulbecco’s medium containing 10% heat-inactivated FBS and the aforementioned antibiotics. The methods were carried out in accordance with the approved guidelines. For transient expression, cells were transfected with expression plasmids using the Lipofectamine 2000 reagent (Invitrogen). For luciferase reporter assays, cells were harvested and analyzed with a commercial kit (Promega). Specific luciferase activities were normalized by protein concentration, which was measured by using the BCA protein assay kit (Pierce, Rockford, IL).

For generating stable knockdown cell lines, BeWo or JEG-3 cells were infected by lentiviral pLKO.1-Puro short-hairpin RNA (shRNA) expression plasmids harboring a scrambled sequence (5′-CCTAAGGTTAAGTCGCCCTCG-3′) or target sequences for GATA3 (5′-CATCCAGACCAGAAACCGAAA-3′) provided by the National RNAi Core Facility of Taiwan. The infected cells were subjected to antibiotic selection using 5 μg/ml of puromycin, and puromycin-resistant clones were pooled for further study.

### Immunohistochemistry and immunofluorescence

To examine GATA3 and GCM1 expression in placenta, early and term human placental tissue biopsy specimens were fixed in 4% paraformaldehyde, dehydrated, embedded in paraffin, and sectioned at 5 μm. Tissue sections were deparaffinized, rehydrated, and subjected to immunostaining by incubation with GCM1, GATA3 (Santa Cruz Biotechnology, Santa Cruz, CA), and cytokeratin 7 (CK7, EMD Millipore, Billerica, MA) antibodies (Abs) overnight, respectively. The sections were then incubated sequentially with biotinylated secondary Ab (Jackson ImmunoResearch, West Grove, PA) and HRP-conjugated streptavidin (Jackson ImmunoResearch). Antigenic detection was performed using DAB peroxidase substrate kit SK-4100 (Vector Laboratories, Burlingame, CA) and the sections were further counterstained with hematoxylin. Co-localization of GCM1 and GATA3 was performed in BeWo and primary human trophoblast cells by co-staining both factors with GCM1 and GATA3 Abs overnight, and then co-incubated with rhodamine-labeled secondary Ab for GCM1 and Cy2-labeled secondary Ab (Jackson ImmunoResearch) for GATA3. Nuclei were stained by DAPI. Immunofluorescence was examined under a Zeiss laser scanning confocal microscope (LSM510).

### Coimmunoprecipitation and pull-down assay

To study the interaction between GCM1 and GATA3 *in vivo*, 293 T cells were transfected with pHA-GCM1 and pGATA3-FLAG. At 48 h post-transfection, cells were harvested in lysis buffer containing 50 mM Tris-HCl (pH 8.0), 150 mM NaCl, 2 mM EDTA, 10% glycerol, 0.5% NP-40, 1 mM DTT, 5 mM NaF, 1 mM Na_3_VO_4_, 1 mM PMSF, and a protease inhibitor cocktail (Sigma-Aldrich, St. Louis, MO), followed by coimmunoprecipitation with FLAG (Sigma-Aldrich) and hemagglutinin (HA, Sigma-Aldrich) Abs. The interaction between endogenous GCM1 and GATA3 was studied in BeWo, JEG-3, and primary human trophoblast cells subjected to coimmunoprecipitation with GCM1 and GATA3 Abs.

To study the interaction between GCM1 and GATA3 *in vitro*, recombinant GATA3-FLAG was first immunopurified with FLAG antibody-conjugated agarose beads (Sigma-Aldrich) from 293 T cells transiently expressing GATA3-FLAG. Recombinant GATA3-FLAG was then incubated with bacterially expressed GST or GST-GCM1, which is a GST fusion protein of GCM1, pre-bound to glutathione-conjugated agarose beads (GE Healthcare Biosciences, Pittsburgh, PA) in lysis buffer at 4 °C for 3 h. After washing three times with lysis buffer, the proteins that were pulled down were analyzed by immunoblotting with FLAG Ab.

To map the GCM1 domain that interacts with GATA3, 293 T cells were transfected with pHA-GATA3 and pGal4-FLAG or pGal4-GCM1-FLAG (the latter contained full-length GCM1 or deletion mutants of GCM1) for coimmunoprecipitation with HA and FLAG Abs. A similar experiment was performed in 293 T cells transfected with pHA-GCM1 and pGal4-FLAG or pGal4-GATA3-FLAG (the latter contained full-length GATA3 or deletion mutants of GATA3) to map the GATA3 domain that interacts with GCM1.

### Chromatin immunoprecipitation (ChIP) analysis and electrophoretic mobility shift assay (EMSA)

ChIP analysis was performed to study the association of GCM1 or GATA3 with HtrA4 promoter in BeWo cells using GCM1 or GATA3 Ab. The primer sequences for ChIP analysis of the GBS in HtrA4 gene are 5′-TGGAAACTGTTACGCTTCTCA-3′ and 5′-GTCTCTAGCCCTACCCG-3′ and the putative GATA3 sites in HtrA4 gene are 5′-GTAGTTGAGGAGTGGAGTTTAGTA-3′ and 5′-GGAGGAGGTCAAACAGTATG-3′ and the promoter region of GAPDH gene are 5′-AAAAGCGGGGAGAAAGTAGG-3′ and 5′-CTAGCCTCCCGGGTTTCTCT-3′.

Direct interaction between GCM1 and the GBS of HtrA4 gene was performed by EMSA as previously described^4^. In brief, a radiolabeled oligonucleotide probe harboring the HtrA4 GBS (5′-CAGTCTGCCCTCATTGTCGG-3′) was incubated with recombinant GCM1-FLAG alone or GCM1-FLAG plus increasing amounts of recombinant GATA3-FLAG. The reaction mixtures were analyzed by electrophoresis on 5% nondenaturing polyacrylamide gels. Recombinant GCM1-FLAG was immunopurified with FLAG antibody-conjugated agarose beads (Sigma-Aldrich) from 293 T cells transiently expressing GCM1-FLAG.

### Quantitative real-time PCR

Cells were lysed using a RealTime ready cell lysis kit (Roche Applied Science, Indianapolis, IN) and reverse transcribed, followed by analysis of GCM1, HtrA4, and GATA3 transcripts in a LightCycler^®^ 480 real-time PCR instrument II (Roche Applied Science). The sequences of the primer sets for PCR analysis are as follows: 5′-CTGACAAGGCTTTTTTCTTCACA-3′ and 5′-CCAGACGGGACAGGTTT-3′ for GCM1, 5′-GACCCACCACCCCATCA-3′ and 5′-GGTTCTGTCCGTTCATTTTGT-3′ for GATA3, 5′-GTCAGCACCAAACAGCG-3′ and 5′-GGAGATTCCATCAGTCACCC-3′ for HtrA4, and 5′-AACTCCATCATGAAGTGTGACG-3′ and 5′-GATCCACATCTGCTGGAAGG-3′ for β-actin.

### Cell invasion assay

Cell invasion analysis of GATA3-knockdown BeWo and JEG-3 cells were performed using Matrigel invasion chambers (BD Biosciences, Bedford, MA) according to the manufacturer’s instructions. In brief, cells were plated into the chambers and incubated for 48 h. Migrated cells in the lower surface of the filters were fixed with paraformaldehyde and visualized by hematoxylin stain and counted. Four microscopic fields per sample were randomly selected for quantification in each of three independent experiments. Images were prepared for presentation using Adobe Photoshop v7.0.

### Statistical analysis

Statistical analysis of the data was performed using Student’s *t* test. Statistical significance was classified as *P < 0.05, **P < 0.01 or ***P < 0.001. A *P* value of <0.05 was considered statistically significant. A *P* value of >0.05 was considered to represent no statistical significance and denoted as “NS”.

## Additional Information

**How to cite this article**: Chiu, Y. H. and Chen, H. GATA3 inhibits GCM1 activity and trophoblast cell invasion. *Sci. Rep.* **6**, 21630; doi: 10.1038/srep21630 (2016).

## Supplementary Material

Supplementary Information
