# Supplementary material for: GATA3 inhibits GCM1 activity and trophoblast cell invasion
Source: Sci Rep. 2016 Feb 22;6:21630. doi: 10.1038/srep21630 (PMC4761948; doi:10.1038/srep21630)
Supplement: Supplementary Information [file srep21630-s1.pdf]

GATA3 inhibits GCM1 activity and trophoblast cell invasion

Yueh Ho Chiu[1,2] and Hungwen Chen[1,2,*]

[1]Graduate Institute of Biochemical Sciences, National Taiwan University, Taipei 106, Taiwan
[2]Institute of Biological Chemistry, Academia Sinica, Nankang, Taipei 115, Taiwan

[*]Address correspondence to: Hungwen Chen, Ph.D., Institute of Biological Chemistry, Academia Sinica, Nankang, Taipei 115, Taiwan, Tel: 886-2-27855696 ext 6090, Fax: 886-2-27889759, E-mail: hwchen@gate.sinica.edu.tw

Supplementary figures 1 to 2

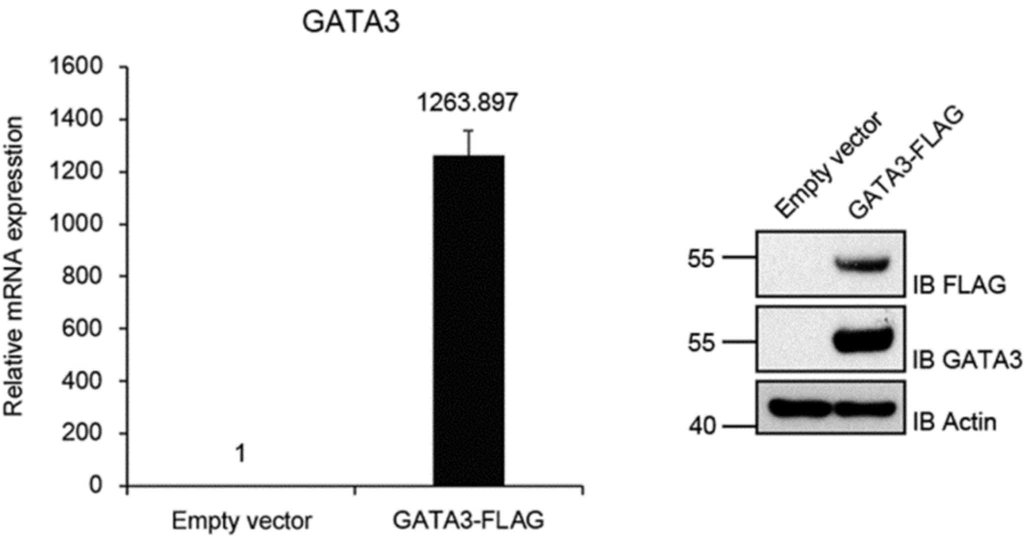

Supplementary Figure 1 Analysis of GATA3 expression in 293T cells. For comparison of endogenous and exogenous GATA3 expression, 293T cells were transfected with 2.5 μg of empty vector or pCDHGATA3-FLAG, which is a CMV promoter-driven expression construct for GATA3 with a C-terminal FLAG tag. At 48 h post-transfection, cells were harvested for measurement of GATA3 and GATA3-FLAG protein and mRNA levels by immunoblotting and quantitative RT-PCR analyses. Only exogenous GATA3-FLAG, but not endogenous GATA3, protein and mRNA could be detected, supporting that 293T cells do not express GATA3. Mean values and the S.D. obtained from three independent experiments are presented.

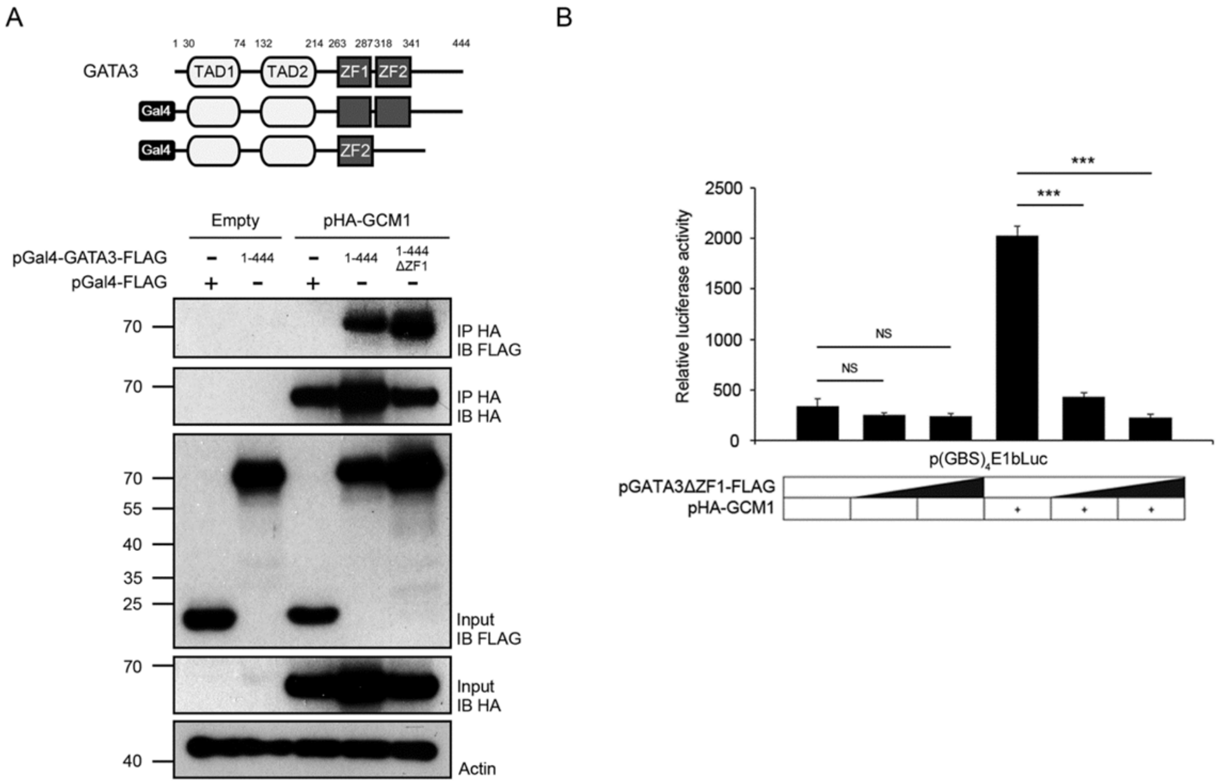

Supplementary Figure 2 Characterization of ZF1-deletion mutant GATA3. (A) ZF1-deletion mutant GATA3 interacts with GCM1. 293T cells were transfected with 2.5 μg of pHA-GCM1 and the indicated pGal4-GATA3-FLAG constructs (2.5 μg) encoding Gal4 fusion proteins with full-length GATA3 or ZF1-deletion mutant GATA3. At 48 h post-transfection, cells were harvested for coimmunoprecipitation assays with HA and FLAG Abs. (B) ZF1-deletion mutant GATA3 suppresses GCM1 transcriptional activity. 293T cells were transfected with 0.05 μg of p(GBS)$_4$E1bLuc, 0.1 μg of pHA-GCM1, and increasing amounts of pGATA3ΔZF1-FLAG (0.1 and 0.3 μg), followed by luciferase reporter assays. Mean values and the S.D. obtained from three independent experiments are presented.
